# Interferon-λ rs12979860 genotype and liver fibrosis in viral and non-viral chronic liver disease

**DOI:** 10.1038/ncomms7422

**Published:** 2015-03-05

**Authors:** Mohammed Eslam, Ahmed M. Hashem, Reynold Leung, Manuel Romero-Gomez, Thomas Berg, Gregory J. Dore, Henry L.K. Chan, William L. Irving, David Sheridan, Maria L. Abate, Leon A. Adams, Alessandra Mangia, Martin Weltman, Elisabetta Bugianesi, Ulrich Spengler, Olfat Shaker, Janett Fischer, Lindsay Mollison, Wendy Cheng, Elizabeth Powell, Jacob Nattermann, Stephen Riordan, Duncan McLeod, Nicola J. Armstrong, Mark W. Douglas, Christopher Liddle, David R. Booth, Jacob George, Golo Ahlenstiel, the International Hepatitis C Genetics Consortium (IHCGC), Javier Ampuero, Margaret Bassendine, Vincent W. S. Wong, Chiara Rosso, Rose White, Lavinia Mezzabotta, Vijayaprakash Suppiah, Monika Michalk, Barbara Malik, Gail Matthews, Tanya Applegate, Jason Grebely, Vincenzo Fragomeli, Julie R. Jonsson, Rosanna Santaro

**Affiliations:** 1 Storr Liver Unit, Westmead Millennium Institute and Westmead Hospital, University of Sydney, Sydney, New South Wales 2145, Australia; 2 Faculty of Engineering, Department of Systems and Biomedical Engineering, Minia University, Minia 6111, Egypt; 3 Institute of Immunology and Allergy Research, Westmead Hospital and Westmead Millennium Institute, University of Sydney, Sydney, New South Wales 2145, Australia; 4 Unit for The Clinical Management of Digestive Diseases and CIBERehd, Hospital Universitario de Valme, Sevilla 41014, Spain; 5 Medizinische Klinik m.S. Hepatologie und Gastroenterologie, Charite, Campus Virchow-Klinikum, Universitätsmedizin Berlin, Berlin 04103, Germany; 6 Department of Hepatology, Clinic for Gastroenterology and Rheumatology, University Clinic Leipzig, Leipzig 04103, Germany; 7 Kirby Institute, The University of New South Wales, Sydney, New South Wales 2052, Australia; 8 St Vincent’s Hospital, Sydney, New South Wales 2052, Australia; 9 Department of Medicine and Therapeutics, Prince of Wales Hospital, The Chinese University of Hong Kong, Hong Kong, China; 10 NIHR Biomedical Research Unit in Gastroenterology and the Liver, University of Nottingham, Nottingham NG7 2UH, UK; 11 Liver Research Group, Institute of Cellular Medicine, Medical School, Newcastle University, Newcastle upon Tyne NE2 4HH, UK; 12 Institute of Translational and Stratified Medicine, Plymouth University, Plymouth PL4 8AA, UK; 13 Division of Gastroenterology and Hepatology, Department of Medical Science, University of Turin, Turin 10126, Italy; 14 School of Medicine and Pharmacology, Sir Charles Gairdner Hospital Unit, University of Western Australia, Nedlands, Western Australia 6009, Australia; 15 Division of Hepatology, Ospedale Casa Sollievo della Sofferenza, IRCCS, San Giovanni Rotondo 71013, Italy; 16 Department of Gastroenterology and Hepatology, Nepean Hospital, Sydney, New South Wales 2747, Australia; 17 Department of Internal Medicine I, University of Bonn, Bonn 53105, Germany; 18 Faculty of Medicine, Medical Biochemistry and Molecular Biology Department, Cairo University, Cairo 11562, Egypt; 19 Department of Gastroenterology and Hepatology, Fremantle Hospital, Fremantle, Western Australia 6160, Australia; 20 Department of Gastroenterology and Hepatology, Royal Perth Hospital, Perth, Western Australia 6000, Australia; 21 Department of Gastroenterology and Hepatology, Princess Alexandra Hospital, Woolloongabba, Queensland 4102, Australia; 22 The University of Queensland, School of Medicine, Princess Alexandra Hospital, Woolloongabba, Queensland 4072, Australia; 23 Gastrointestinal and Liver Unit, Prince of Wales Hospital and University of New South Wales, Sydney, New South Wales 2031, Australia; 24 Department of Anatomical Pathology, Institute of Clinical Pathology and Medical Research (ICPMR), Westmead Hospital, Sydney, New South Wales 2145, Australia; 25 School of Mathematics and Statistics, University of Sydney, Sydney, New South Wales 2006, Australia; 26 The Kirby Institute, University of New South Wales, Sydney, New South Wales 2033, Australia.; 27 List of members and affiliations appears at the end of the paper

## Abstract

Tissue fibrosis is a core pathologic process that contributes to mortality in ~45% of the population and is likely to be influenced by the host genetic architecture. Here we demonstrate, using liver disease as a model, that a single-nucleotide polymorphism (*rs12979860)* in the intronic region of interferon-λ4 (IFNL4) is a strong predictor of fibrosis in an aetiology-independent manner. In a cohort of 4,172 patients, including 3,129 with chronic hepatitis C (CHC), 555 with chronic hepatitis B (CHB) and 488 with non-alcoholic fatty liver disease (NAFLD), those with rs12979860CC have greater hepatic inflammation and fibrosis. In CHC, those with rs12979860CC also have greater stage-constant and stage-specific fibrosis progression rates (*P*<0.0001 for all). The impact of rs12979860 genotypes on fibrosis is maximal in young females, especially those with HCV genotype 3. These findings establish rs12979860 genotype as a strong aetiology-independent predictor of tissue inflammation and fibrosis.

## Abstract (web summary)

Tissue fibrosis is a major contributor to mortality in the developed world. Here, the authors identify a genetic variant in the intronic region of interferon-λ4 that is a strong predictor of hepatic inflammation and fibrosis, independent of liver disease aetiology

## Graphical abstract

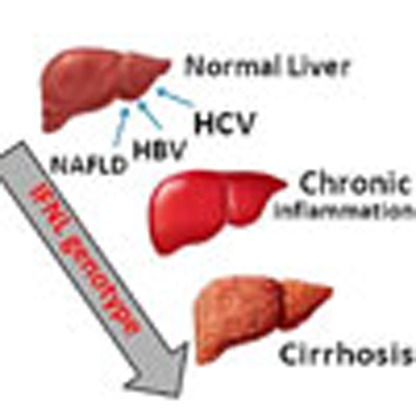

Chronic fibro-proliferative diseases account for ~45% of all deaths in the developed world^1^. End-stage liver disease is a *sine qua non* of this phenomenon, as fibrosis culminating in cirrhosis is the principal cause of liver-related morbidity and mortality^2^. There are probably ‘core’ and ‘regulatory’ pathways, as well as susceptibility genes, that play a role in fibrosis evolution and they are likely aetiology independent^3^; however, these have yet to be defined.

Liver disease develops as a consequence of any number of insults, with chronic viral hepatitis B and C (CHB and CHC) and non-alcoholic fatty liver disease (NAFLD) among the most prevalent. These three diseases therefore offer an opportunity to understand the role of genetic factors in the evolution of liver fibrosis and to determine whether they are aetiology independent.

Emerging evidence suggests that host genetics influence liver fibrosis, particularly variants in genes controlling the immune and inflammatory response pathways^4^,^5^,^6^. In this context, the discovery of polymorphisms (*rs12979860* and *rs8099917)* in the interferon-λ (IFN-λ) region, represented a breakthrough in hepatitis C research, being the strongest host factor associated with viral clearance after IFN-based therapy^7^,^8^,^9^,^10^. As the *rs12979860* is located within intron 1 of the newly discovered IFN-λ4 (*IFNL4*) gene^11^, *rs12979860* is here referred to as an *IFNL4* single-nucleotide polymorphism (SNP).

*rs12979860* ‘responder’ genotypes have been shown in several reports to be associated with greater hepatic inflammation^12^,^13^,^14^. However, whether *rs12979860* genotype predicts liver fibrosis progression is controversial, in particular for diseases other than CHC^12^,^13^,^14^,^15^,^16^,^17^,^18^,^19^,^20^,^21^. A recent study added to this controversy by demonstrating that although CHC patients with the *rs12979860* CC responder genotype had greater hepatic necroinflammation and worse clinical outcomes, they had lower mean Ishak fibrosis scores, compared with those with *rs12979860* CT/TT genotypes, and no association with fibrosis progression on paired biopsies^13^. These findings are hard to reconcile, as fibrosis is a consequence of hepatic inflammation^1^,^22^ and prospective paired biopsy cohorts clearly demonstrate that necroinflammatory grade on initial liver biopsy is the best predictor of fibrosis progression^23^,^24^. A recent meta-analysis failed to resolve this conundrum, as although the authors demonstrated that *rs12979860* CC and *rs8099917* TT genotypes were not associated with severe inflammation in treatment–naive patients, *rs12979860* CC was only weakly associated with fibrosis (*P*=0.04)^25^, probably reflecting the inter-study variability in the outcomes.

In the present study we sought to clarify the impact of polymorphisms in *rs12979860* on the progression of liver inflammation and fibrosis in a large cohort of patients with CHC; to explore the interactions of *rs12979860* genotype with host and viral factors including age, gender and viral genotype that might modulate this effect; and to explore the impact of *rs12979860* genotype on liver fibrosis in other viral (CHB) and non-viral (NAFLD) liver diseases. We demonstrate that *IFNL* genotype is a strong predictor of hepatic inflammation and fibrosis, independent of disease aetiology, and provide evidence that this risk is modulated by clinical factors (gender, age and HCV genotype).

## Results

### Patient characteristics

The study comprises 4,172 patients including 3,129 with CHC, 555 with CHB and 488 with NAFLD. The genotype distribution of the two *IFNL* SNPs (*rs12979860* and *rs8099917*) was in Hardy–Weinberg equilibrium (Supplementary Table 1). For CHC, 3129 Caucasian patients were enroled. The baseline demographic, laboratory and histologic data are shown in Supplementary Table 2.

For the CHC cohort, the median age was 44 years, 63% were male, 73% were infected with HCV genotype 1 and 14% had heavy alcohol consumption (≥50 g daily). With regard to rs12979860 genotypes, 36% of patients had CC genotype, 49% CT and 15% TT. Patients (49.4%) had significant fibrosis (Metavir score F2–4), 43.3% had moderate/severe necroinflammation (Metavir A2–A3) and 18.2% had moderate–severe steatosis.

### Choice of the genetic model

The co-dominant and dominant models were similarly able to adequately address the association between *rs12979860* and liver fibrosis, regardless of aetiology. The dominant model was the most appropriate, as it had the lowest Akaike information criterion (AIC) value (Supplementary Table 3) Thus, all results are presented using the dominant model.

### *IFNL* genotype hepatic inflammation and fibrosis in CHC

**Figure 1:**
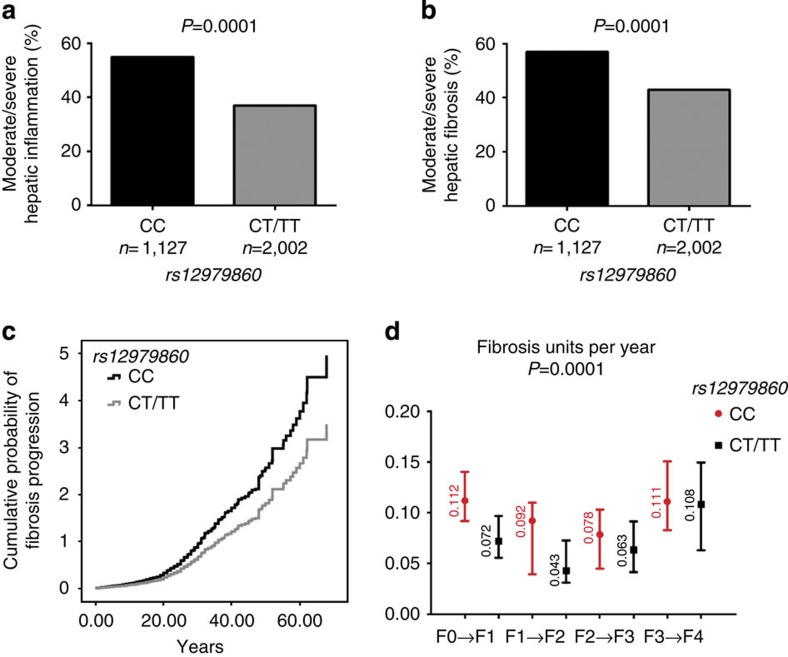
IFNL genotype and hepatic inflammation and fibrosis. (**a**) rs12979860 genotype and hepatic inflammation degree in the cohort of patients with chronic hepatitis C (*n*=3,129). Pearson’s *χ*^2^-test and Fisher’s exact test were used to compare hepatic inflammation rates. (**b**) rs12979860 genotype and liver fibrosis stage in the cohort of patients with chronic hepatitis C (*n*=3,129). Pearson’s *χ*^2^-test and Fisher’s exact test were used to compare hepatic fibrosis rates. (**c**) Multivariate cox regressions analysis of rs12979860 genotype on the cumulative probability of progression to moderate/severe (≥F2) fibrosis after adjusting for covariates (age, gender, BMI, duration of the infection, HCV genotype, inflammation progression and basal ALT, AST, GGT, platelets, bilirubin and alkaline phosphatases) in 1,312 patients with an estimated duration of HCV infection. Bars indicate 95% CIs. (**d**) Stage-specific rates and 95% CIs of fibrosis progression according to rs12979860 genotype in 1,312 patients with an estimated duration of HCV infection. FPRs were obtained using the Markov maximum likelihood estimation. *P*-values were obtained using a likelihood ratio test comparing models with and without rs12979860 genotype. FPRs were significantly increased for the rs12979860 CC genotype compared with rs12979860 non-CC (*P*=0.0001). The influence of rs12979860 CC genotype was more important for early compared with late fibrosis stage transitions. Bars indicate 95% CIs.

By multiple logistic regression analysis, Subjects with *rs12979860* CC had higher necroinflammatory activity (Fig. 1a; odds ratio (OR): 2.11, 95% confidence interval (CI): 1.82–2.45; *P*<0.0001). Subjects with *rs12979860* CC also had higher significant fibrosis (≥F2) (Fig. 1b; OR: 1.63, 95% CI: 1.24–2.51; *P*<0.0001). Univariate and multivariate analysis of factors associated with fibrosis in cross-sectional analysis (*n*=3,129) is shown in Table 1. In univariate analysis, apart from *rs12979860*, significant fibrosis was associated with the previously reported risk factors of older age, male sex, higher body mass index (BMI), higher alanine aminotransferase (ALT), aspartate aminotransferase (AST), alkaline phosphatase, γ-glutamyl transpeptidase (GGT), bilirubin and lower platelet counts, infection with HCV genotype 3 and with moderate–severe hepatic inflammation. In multivariate analysis, rs12979860, age, sex, AST, GGT, platelets, HCV genotype 3 and hepatic inflammation remained independently associated with significant fibrosis. Alcohol was not associated with fibrosis (Table 1). We further investigated the association of alcohol use categorized as quintiles; this analysis also demonstrated no significant association with liver fibrosis. As data regarding alcohol intake are self-reported, the possibility of lack of standardization cannot be excluded. HCV genotype 1 was not associated with significant fibrosis (*P*=0.2). Further sub-categorization was not feasible, owing to the small numbers with genotype 2 and 4.

### Association of clinical factors with the rs12979860 genotype

As previously reported^12^,^14^, patients with *rs12979860* CC genotype had higher median ALT, AST, GGT, bilirubin level and lower platelet counts compared with non-CC genotypes (*P*<0.001 for all) at the time of liver biopsy (Supplementary Table 4). Patients with rs12979860CC genotype were also more likely to be infected with HCV genotype 3 than the CT and TT genotypes (51.7% versus 48.3%, OR: 1.78, 95% CI: 1.28–2.48; *P*=0.0001).

### *IFNL* genotype hepatic inflammation and fibrosis progression

The baseline characteristics were similar among subjects included and not included in the fibrosis progression analysis sub-population (that is, estimated duration of infection, *n*=1,312). The proportion of patients with a rapid fibrosis progression rate (FPR) (that is, higher than the median) was higher for *rs12979860* CC than *rs12979860* CT/TT (58% versus 42%; *P*=0.0001). This association remained independently predictive in a multiple logistic regression model (OR: 1.58, 95% CI: 1.26–1.99; *P*<0.0001; Supplementary Table 5).

As fibrosis progression may not be constant over time^22^ and FPR assumes linearity, complementary analytical approaches were undertaken to avoid this source of bias. Using Cox proportional hazards, *rs12979860* CC remained independently associated with an increased hazard of progression to significant fibrosis (adjusted hazards ratio: 1.41, 95% CI: 1.21–1.64; *P*<0.0001; Fig. 1c). We additionally computed stage-specific progression rates using the Markov maximum likelihood estimation. Again, the *rs12979860* genotype remained predictive of fibrosis progression (*P*<0.0001). Interestingly, the effect on fibrosis of the rs12979860 CC genotype was stronger at early fibrosis transitions (F0 to F1 and F1 to F2) compared with later stages (F2 to F3 and F3 to F4; Fig. 1d).

To further confirm the association of *rs12979860* genotype with fibrosis, we assessed 106 patients with paired liver biopsies. In this cohort, the median duration between biopsies was 6 years (characteristics in Supplementary Table 6) and there was greater progression in inflammation (≥2 points) and fibrosis (any progression and ≥2 points) in the responder genotypes of both *rs12979860* and *rs8099917* SNPs, whereas the association with FPR and the proportion of patients with any or ≥2 points fibrosis progression was significant only for *rs8099917* (Supplementary Table 7). Overall, similar results were observed with the responder variant of *rs8099917* for all analyses.

*In toto*, these results demonstrate that responder *rs12979860* and *rs8099917* genotypes are associated with increased liver fibrosis and with more rapid fibrosis progression in CHC.

### Interaction of rs12979860 with fibrosis risk factors

**Figure 2:**
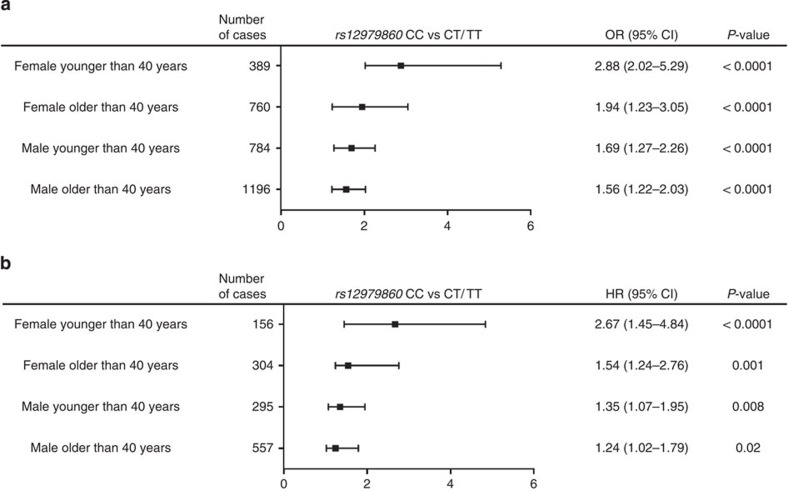
Interaction of rs12979860 with age and gender. (**a**) Multiple logistic regression analysis of rs12979860 genotype on the OR of having moderate/severe (≥F2) fibrosis stratified according to age and gender in the cohort of patients with chronic hepatitis C (*n*=3,129). Bars indicate 95% CIs. (**b**) Multivariate Cox regression analysis of rs12979860 genotype on the hazards of having moderate/severe (≥F2) fibrosis stratified according to age and gender in 1,312 patients with an estimated duration of HCV infection. Bars indicate 95% CIs.

Using stratification analysis, we examined the association of *rs12979860* genotype with significant fibrosis by different known risk factors for fibrosis progression (that is, age, sex, alcohol consumption, ALT (a surrogate marker of necro-inflammation) and HCV-genotype) categories. As shown in Table 2, the results for *rs12979860* genotype were significantly more prominent in younger (<40 years) females and those with HCV genotype 3 infection compared with younger (<40 years old) males with non-genotype 3 infection. As age in general and reproductive age in females are established risk factors for fibrosis^26^,^27^, we stratified the gender association according to age. Interestingly, the effect of gender was more prominent in younger (<40 years) compared with older females (OR: 2.88, 95% CI: 2.02–5.29 versus OR: 1.94, 95% CI: 1.23–3.05; *P*=0.01) and to a less extent in younger (<40 years) compared with older males (OR: 1.69, 95% CI: 1.27–2.26 versus OR: 1.56, 95% CI: 1.22–2.03; *P*=0.6 and *P*=0.001 for trend) (Fig. 2a).

To further confirm this observation, we explored by stratification analyses the associations of *rs12979860* genotype in the fibrosis progression sub-cohort (that is, estimated duration of infection). Overall, as reported previously, women had slower fibrosis progression than men (median FPR was 0.071 versus 0.087; *P*=0.01). The association of *rs12979860* CC with fibrosis progression was more prominent in younger (<40 years) compared with older females and younger versus older males (*P*=0.001 for trend; Fig. 2b).

In addition to the stratified analysis, there was also evidence of significant multiplicative interaction with sex and age. No statistically significant multiplicative interaction was found between *rs12979860* genotype and HCV genotype 3. After allowance for multiple testing, ALT and alcohol consumption yielded no significant evidence of an interaction with rs12979860 genotypes (Table 2).

Lastly, we investigated the joint effect of *rs12979860* genotype with age and sex. There was further evidence of interaction with sex, as males with *rs12979860* CC had an increased OR for significant fibrosis (OR: 2.53, 95% CI: 2.28–2.85; OR: 1.4, 95% CI: 1.16–1.68; and OR: 1.73, 95% CI: 1.52–2.23; *P*=0.002 for trend), compared with the reference group (females without CC genotypes, females with CC genotypes and males without the CC genotype, respectively). Of note, the distribution of the *rs12979860* SNP and the median duration of infection until time of biopsy were not significantly different between males and females.

We also demonstrated a joint effect of *rs12979860* genotype and age at time of biopsy. Thus, subjects over 40 years old with *rs12979860* CC had an increased OR of significant fibrosis (OR: 3.91, 95% CI: 3.13–4.9; OR: 2.35, 95% CI: 1.94–2.85; OR: 1.79, 95% CI: 1.4–2.28, respectively; *P*<0.0001 for trend) compared with the reference group (younger than 40 years old without CC genotype, younger than 40 years old with CC genotype and over 40 years old without the CC genotype, respectively).

Using logistic regression, a striking interaction between the *rs12979860* SNP, age and gender was observed. Males over 40 years with *rs12979860* CC had an increased OR for significant fibrosis (OR: 7.58, 95% CI: 5.52–9.38) compared with the reference group (female subjects younger than 40 years old with non-CC genotypes; *P*<0.0001). Overall, similar results were observed with *rs8099917*.

Taken together, the *rs12979860* genotype demonstrates interactions with age and sex in mediating liver fibrosis, being more obvious in young females, but also demonstrating interaction with the classic risk factors (older age and male gender) in increasing fibrosis.

### Association of rs12979860 with fibrosis in other diseases

**Figure 3:**
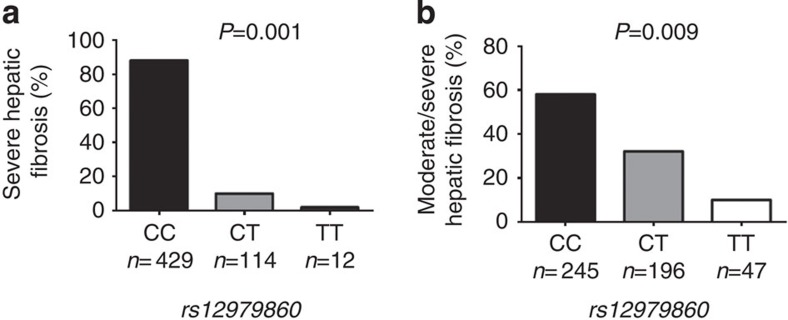
IFNL genotype and fibrosis in other non-CHC diseases. rs12979860 genotype and liver fibrosis in patients with (**a**) CHB (*n*=555) and (**b**) NAFLD (*n*=488). Pearson’s *χ*^2^-test and Fisher’s exact test were used to compare hepatic fibrosis rates.

*CHB cohort*. The baseline demographic, laboratory and histologic data are shown in Supplementary Table 8. After adjustment for age, ethnicity, HBV-DNA levels, liver enzymes and HBe-Ag status, *rs12979860* CC was associated with hepatic inflammation (OR: 4.36, 95% CI: 2.46–7.74; *P*<0.0001). Likewise, *rs12979860* CC was associated with severe fibrosis (OR: 2.75, 95% CI: 1.23–6.14; *P*<0.001; Table 3 and Fig. 3a).

*NAFLD cohort*. The baseline demographic, laboratory and histologic data for this cohort are shown in Supplementary Table 9. To examine whether the association of *rs12979860* genotypes with the histological severity of NAFLD is independent of the well-known risk factors for liver damage in NAFLD, we considered in the analysis cardio-metabolic risk factors such as diabetes, hypertension, lipid profiles and homeostasis model assessment-estimated insulin resistance (HOMA-IR).

Univariate predictors of moderate–severe liver fibrosis in NAFLD are presented in Table 4 and Fig. 3b. *rs12979860* CC was associated with both portal and lobular inflammation (OR: 3.5, 95% CI: 1.81–6.61 and OR: 2.65, 95% CI: 1.5–4.68; *P*<0.0001 for both). By logistic regression, apart from *rs12979860* CC, older age, high insulin, high blood glucose, high HOMA-IR, low platelet counts, AST, GGT, ALT, the presence of hypertension, the presence of diabetes and portal inflammation were associated with significant fibrosis. In a model excluding hepatic inflammation owing to its strong association with *rs12979860* genotype, *rs12979860* CC was associated with significant fibrosis (OR: 1.66, 95% CI: 1.15–2.43; *P*=0.006; Table 4).

Taken together, rs12979860 genotypes associate with liver injury (hepatic inflammation) and fibrosis, independent of the known risk factors.

## Discussion

The association between *rs12979860* and liver fibrosis has been controversial. Herein we provide strong evidence in large cohorts and using multiple complementary approaches that *rs12979860* genotype is associated with hepatic inflammation and fibrosis, irrespective of disease aetiology. We have confirmed previous data that host and viral factors modulate fibrosis risk, and now provide additional evidence for an independent role for the *rs12979860* polymorphism in modulating the phenotype. Of particular interest, we can demonstrate that the genetic effects on fibrosis are maximal in young females, a group traditionally considered to have a low risk of CHC disease progression.

Using a comprehensive validation strategy (large patient cohort, a subset with estimated duration of infection and multiple analytical approaches) in both cross-sectional and longitudinal studies, *rs12979860* genotype was unequivocally associated with inflammation and fibrosis stage, and with faster FPRs and fibrosis progression in paired biopsies. Consistent with the notion from other cohorts with dual liver biopsies^23^,^24^ that inflammation is a dominant driver of fibrosis, patients with the responder genotypes also had elevated aminotransferases and more inflammation on liver biopsy^28^. The discovery of *rs12979860* genotypes as a predictor of HCV clearance represented a milestone for decision-making with regard to IFN-based treatment. The present data now suggest that *rs12979860* genotyping is also highly informative in staging fibrosis and in predicting the rate of liver fibrosis progression.

Several studies have examined the association between *rs12979860* and *rs8099917* genotypes and liver histology with conflicting results^12^,^13^,^14^,^15^,^16^,^17^,^18^,^19^,^20^,^21^. In most reports^12^,^13^,^14^,^21^, the *rs12979860* genotype appears to be associated with hepatic inflammation (as we have confirmed), while effects on fibrosis have been harder to demonstrate. Some studies in fact have shown no association at all or an association of the minor *rs12979860* genotype TT (or *rs8099917* GG) with more advanced fibrosis or cirrhosis^13^,^14^,^15^,^16^,^17^,^18^. Only two studies have demonstrated an association mainly for non-genotype 1 (refs 12, 19) with the *rs8099917* responder genotype. A recent publication added controversy by demonstrating that although CHC patients with *rs12979860* CC had greater necroinflammation and worse clinical outcomes, they had lower mean Ishak fibrosis scores, compared with those with *rs12979860* CT/TT genotypes, and no difference in fibrosis progression in paired biopsies^13^.

We suggest that the principal reason for the discrepant results is the limitation of statistical power in the earlier reports. In contrast to the present study that included >3,100 patients with CHC, prior reports usually had <400 patients^15^,^16^,^17^. To date, the two largest reported cohorts with histology had 1,019 and 1,483 patients^12^,^13^. In these, differences in patient characteristics might have additionally accounted for the variance in the results. For example, to demonstrate an effect on liver fibrosis progression, the study by Noureddin *et al*.^13^ included individuals who had received a prior course of therapy. This might have influenced their results as a recent report based on paired biopsy analysis suggests that treatment failure might accelerate fibrosis progression in CHC^29^. Inclusion of those with treatment failure can also lead to selection bias, as patients with *rs12979860 CC* with milder stages of fibrosis may have preferentially responded to therapy, thereby enriching the cohort with *rs12979860* non-CC subjects with more severe liver fibrosis. In the present study, all analyses only included patients that had not received prior therapy.

We demonstrated significant interactions of *rs12979860* genotype with age and gender on the stage of liver fibrosis. Of interest, the influence of the *rs12979860* responder genotype on fibrosis was greater in women than men. This was not due to differences in the frequencies of rs12979860 genotypes, the duration of infection or the distribution of viral genotypes or viral load. This result is to be expected given that *rs12979860* genotype, as a hazard variation, would exert its effect most profoundly in individuals less likely to develop fibrosis (that is, women). In other words, women who eventually develop fibrosis are more likely to be genetically predisposed to it. Along the same vein, we have shown that the influence of the *rs12979860* responder genotype on fibrosis is greater in younger than in older women. We also demonstrated that the *rs12979860* genotype demonstrates synergy with the classic risk factors (older age and male gender) in increasing fibrosis. At the individual patient level, it suggests that early anti-viral treatment should be considered for those at highest genetic risk of rapid fibrosis, that is, those with *rs12979860* responder genotypes. These findings also have implications for drug trials of anti-fibrotic therapies, requiring proper stratification to avoid type 2 errors, or restricting enrolment only to those at highest risk, to demonstrate an effect.

We have confirmed that HCV genotype 3 is associated with more advanced fibrosis than non-genotype 3 (ref. 30). A recent study also indicated an approximately twofold increase in all-cause mortality and Hepatocellular carcinoma (HCC) in patients with genotype 3 as compared with non-genotype 3 (ref. 31). Interestingly, we observed that the effect of *rs12979860* responder status on fibrosis stage was more profound in genotype 3. In addition, those with *rs12979860* CC were more likely to be infected with genotype 3 than with non-genotype 3, as previously suggested^32^. The latter is consistent with our observation that HCV genotype 1 is associated with greater spontaneous clearance compared with non-genotype 1 (ref. 33). This leads us to speculate that *rs12979860* genotype might play a causal role in linking reduced spontaneous clearance, accelerated fibrosis progression and increased overall mortality and HCC in genotype 3 CHC.

We observed that the effect of *rs12979860* responder genotypes was more important in early (F0 to F1 and F1 to 2) rather than late (F2 to F3 and F3 to F4) fibrosis transitions. This suggests additional complexity in genetic effects. There is experimental evidence for this. Natural killer (NK) cells promote a proinflammatory milieu in the early stages of fatty liver disease^34^, and *IFNL* and *HLA-C* genotypes likewise interact to influence natural killer activity, in particular during early infection^35^. However, the validity of our observation requires verification in independent cohorts.

We have demonstrated that *rs12979860* genotype influences necroinflammation and fibrosis in both CHB and in NAFLD. This observation is novel and emphasizes the fact that common susceptibility genes may play a role in fibrosis evolution, independent of disease aetiology. To the best of our knowledge, ours is the first report showing that *rs12979860* polymorphisms have an association with liver histology in CHB. Only two studies to our knowledge have addressed the role of *rs12979860* polymorphisms in NAFLD, both contradictory^20^,^21^. The cohort sizes in these reports ((*n*=160, showing an association with inflammation and fibrosis^21^, and *n*=196, showing no effect on histology^20^) probably accounts for the divergent results. Our larger cohorts therefore help to clarify the role of rs12979860 in liver fibrosis in these conditions, in particular the context of our consistent overall story.

It could be questioned as to why several apparently relevant genome-wide association study (GWAS) of HCV- and HBV-related HCC^36^,^37^, which occur in the context of cirrhosis, or others on HCV treatment outcomes in which liver histology was available^7^,^8^,^9^ have failed to identify associations between IFN-λ region genetic variants and liver fibrosis. However, this may be methodological as highlighted by recent criticisms of the utility of GWAS that focus on susceptibility to inform on other aspects of a disease^38^. For example, a recent paper in *Cell*, leveraging from a GWAS on susceptibility, identified a non-coding polymorphism in FOXO3A not identified by the GWAS (rs12212067: T>G) that associated with the course of, but not susceptibility to, Crohn’s disease and rheumatoid arthritis, and with an increased risk of severe malaria^39^. Hence, GWAS design (for example, of susceptibility versus progression) is critical to outcome.

Recently, several functional variants have been discovered in the IFN-λ region, including *IFNL4*. Production of the IFNL4 protein is dependent on the ΔG/TT (*rs368234815*) frameshift polymorphism^11^,^40^ and predicts HCV clearance better than the *rs12979860* SNP. A second polymorphism in the 3′-untranslated region region of *IFNL3* (*rs4803217*) affects the messenger RNA stability of IFNL3 (ref. 41). We also identified a third polymorphism, *rs4803221*, which predicts Sustained virologic response (SVR) better than *rs12979860* (ref. 42). Although the role of these variants in predicting liver fibrosis is unknown, especially in non-HCV cohorts, they are in strong linkage disequilibrium (LD) with *rs12979860* and, therefore, we speculate will provide similar results.

Although the mechanisms for the fibrogenic effect of *rs12979860* genotypes are beyond the scope of the current work, some hypotheses can be entertained. For example, Sheahan *et al*.^43^ recently demonstrated that in human hepatocytes (HCV infected and non-infected), cell death- and survival-related networks were the top upregulated pathway in responder rs12979860 genotype patients. In that study, donors with responder genotypes were able to generate more effective anti-viral immune responses compared with those with the non-responder genotype. Along the same vein, Raglowet *et al*.^44^ demonstrated higher basal interferon-stimulated genes (ISGs) in non-infected livers of *rs12979860* responder patients. Thus, it is possible that *IFNL* genotype, by activation of immune, cell death and survival responses, could on the one hand render hepatocytes more efficient at responding to infection, but on the other, drive hepatic inflammation and liver fibrosis.

In conclusion, we provide strong evidence that the *IFNL* genotype modulates hepatic inflammation and fibrosis. This effect is independent of disease aetiology and in the case of hepatitis C is independent of, but synergistic with, age and gender. Understanding the biological basis for these observations will be the next important step, but in the meantime our data provide an evidence base for the development of individualized patient management algorithms.

## Methods

### Patient cohort

The study comprises 4,172 patients, including 3,129 with CHC, 555 with CHB and 488 with NAFLD. For CHC, 3,129 Caucasian patients were from the International Hepatitis C Genetics Consortium database of chronically infected HCV patients. The International Hepatitis C Genetics Consortium included patients from 16 tertiary and academic centres from 5 countries (Australia, United Kingdom, Spain, Italy and Germany). All consecutive patients at these centres, who had a liver biopsy with scoring for fibrosis stage and disease activity before treatment between 1999 and 2011, were included. Patients were excluded if they had evidence of other liver diseases by standard tests. Additional fibrosis progression analyses were conducted in a subset of 1,312 patients from the initial cohort with an estimated duration of infection, which enabled calculation of the FPR per year. A further 106 patients with paired liver biopsies were evaluated; none in this cohort had cirrhosis at the initial biopsy and had the subsequent biopsy a minimum of 1 year and a maximum of 10 years later. The second biopsy was undertaken at the discretion of the treating clinician as part of routine clinical care and no patient had received IFN-based therapy either before the initial biopsy or between the first and second biopsies. Ethics approvals were obtained from the following Human Research Ethics Committees: the Sydney West Area Health Service, the University of Sydney, Human Research Ethics Committee; South Eastern Sydney Local Health District, NSW Health; Ethics Committee of St Vincent’s Hospital, Sydney; Nepean Blue Mountains Local Health District Human Research Ethics Committee, Sydney; The Royal Perth Hospital Human Research Ethics Committee, WA; Metro South Human Research Ethics Committee, QLD; Human Research Ethics Committee, South Metropolitan Health Service, Fremantle, WA; Sir Charles Gairdner Hospital Human Research Ethics Committee, WA; Ethics Committee of Valme University Hospital, Seville; Ethics Committee of Medical Research of the University of Leipzig, Berlin; Ethics Committee of Medical Research the University of Berlin (Charité), Berlin; Northern & Yorkshire MREC, Nottingham; Ethical Committee of the Città della Salute e della Scienza University Hospital, Torino; Ethical Committee IRCCS ‘Casa Sollievo della Sofferenza’ Hospital San Giovanni Rotondo, Italy; Joint Chinese University of Hong Kong–New Territories East Cluster Clinical Research Ethics Committee, Hong Kong; Northumberland Research Ethics Committee, Newcastle; and the Ethics Committee of the Medical Faculty, Rheinische Friedrich-Wilhelms University Bonn, Bonn. All patients provided written informed consent.

### Clinical and laboratory assessment

The following data were collected at the time of liver biopsy from all patients: sex, age, ethnicity, recruitment centre, alcohol intake, BMI and routine laboratory tests. Alcohol consumption was assessed by two separate interviews with the patient and close family members. BMI was calculated as mass divided by the square of the height (kg m^−2^). Data on cardio-metabolic status were collected from the NAFLD cohort. The diagnosis of arterial hypertension and diabetes was according to international criteria^45^. ALT, AST, γ-glutamyl transferase, prothrombin time, bilirubin, haemoglobin, leukocyte and platelet count were determined by routine laboratory techniques. After an overnight fast, venous blood was drawn to determine serum levels of triglyceride, high-density lipoprotein cholesterol, insulin and C-peptide. Serum insulin and C-peptide were determined by routine laboratory techniques. IR was determined by the HOMA method using the following equation: HOMA-IR=fasting insulin (mU ml^−1^) × fasting plasma glucose (mmol l^−1^)/22.5.

### Methods to estimate the duration of infection

Fibrosis progression was examined in 1,312 CHC patients with a reliable estimated duration of infection. For patients with a history of blood transfusion (*n*=371), the onset of infection was assumed to be the year of transfusion. For subjects with a history of injecting drug use (*n*=749), the time of infection was estimated using the reported ‘first year of injection’. For patients with a history of occupational exposure (*n*=192), the onset of infection was assumed to be the first year of a needle stick exposure. The duration of infection was calculated by subtracting the estimated age at infection from age at biopsy.

### Liver histopathology

Biopsies were interpreted by a single expert liver pathologist in each centre, who was blinded to patient clinical characteristics, serum measurements and *IFNL* genotyping. The interobserver agreement between pathologists was studied previously and was good for METAVIR staging using *κ* statistics (*κ*=77.5)^10^. All biopsies had a minimum length of 15 mm or the presence of at least 11 complete portal tracts; inadequate biopsies were excluded^46^. Liver histopathology for cases with CHC or CHB was according to METAVIR^47^. Fibrosis was staged from F0 (no fibrosis) to F4 (cirrhosis). Necroinflammation (A) was graded as A0 (absent), A1 (mild), A2 (moderate), or A3 (severe). For NAFLD, the Kleiner classification was used to compute the NAFLD activity score (from 0 to 8, on a scale including separate scores for steatosis, lobular inflammation and hepatocellular ballooning) and to stage fibrosis from 0 to 4 (ref. 48).

### Genotyping

Genotyping for *rs12979860* and *rs8099917* SNPs was undertaken using the TaqMan SNP genotyping allelic discrimination method (Applied Biosystems, Foster City, CA, USA). The *rs8099917* genotyping kit was supplied by Applied Biosystems and rs12979860 genotyping was performed using a custom-designed genotyping assay from Applied Biosystems. Genotyping was performed using the StepOne RT system and analysed with StepOne software v.2.3.0 (Applied Biosystems). All genotyping was undertaken blinded to clinical variables.

### Statistical analysis

Statistical analyses were performed using the statistical software package SPSS for Windows, version 21 (SPSS, Chicago, IL), SAS version 9.1 and SAS Enterprise 9.4, unless otherwise indicated. Results are expressed as mean±s.d. or number (percentage) of patients. Student’s *t*-tests or non-parametric tests (Mann–Whitney *U*-test) were used to compare quantifiable data, as appropriate. The *χ*^2^- and Fisher’s exact tests were used for comparison of frequency data and to evaluate the relationships between groups. All tests were two-tailed and *P*-values <0.05 were considered significant.

Multiple logistic regression models were used to assess for factors independently associated with liver injury. For this analysis, necroinflammation was dichotomized as absent/mild (Metavir score A0–A1) or moderate/severe (Metavir score A2–A3) and fibrosis as absent or mild (Metavir score F0–1), or significant (Metavir score F2–4). For the NAFLD cohort, factors independently associated was portal inflammation dichotomized as absent–mild (portal inflammation 0–1) or moderate–severe (portal inflammation 2–3), and fibrosis dichotomized as absent/mild (F0–F1) or significant (F2–F4) was determined. The OR estimates the relative change in the rate of the outcome (significant fibrosis) per unit increase in the explanatory variable. Apart from rs12979860, the other covariates included known liver fibrosis risk factors such as age, sex, route of infection, serum HCV RNA level, HCV genotype, BMI, diabetes, the grade of METAVIR activity, alcohol consumption, liver enzymes, platelets count and recruitment centre as well. Variables with *P*<0.20 in univariate analyses with the outcome of interest were included in multivariate analyses.

We examined five potential genetic models that might explain the effect of *rs12979860* on liver fibrosis: co-dominant, dominant, recessive, over dominant and log additive using SNPStats (http://bioinfo.iconcologia.net/index.php)^49^,^50^. The Log Additive model compares major allele homozygotes versus heterozygotes versus minor allele homozygotes, presuming an *r*-fold risk for heterozygote subjects and a 2*r*-fold risk for homozygous subjects with the major allele. We investigated which model was the most appropriate by calculating the AIC values^51^. The lowest AIC is indicative of the best fit. The strength of the association between rs12979860 and significant fibrosis under each model was expressed by ORs and their corresponding 95% CIs.

The FPR in the 1,312 Caucasians patients with CHC and an estimated duration of infection was determined by calculating the ratio between the fibrosis stage and the estimated disease duration (in years). Three approaches were used to assess fibrosis progression. Patients were stratified into two groups of stage-constant FPRs according to the median rate (that is, 0.076 fibrosis units per year), which was used as a cutoff. Factors associated with rapid FPR (that is, higher than the median) were analysed by univariate and multivariate regression analysis. Two additional approaches were used to assess fibrosis progression and to confirm the results of the multivariate regression. First, an estimation of the stage-specific progression rate using a Markov maximum likelihood estimation method, as suggested by Yi *et al*.^52^, was applied to estimate the annual stage-specific transition probabilities (for example, F0 to F1, F1 to F2 and so on). Second, we used Cox regression analysis to model the time taken for significant fibrosis to occur. For this, after checking the normality of the quantified variables, appropriate logarithmic transformations were made. We considered estimated age at infection as the starting point and the first liver biopsy showing significant fibrosis (failure time) or the last liver biopsy showing an absence of significant fibrosis in the absence of treatment (censored time) as the end point. A Cox proportional-hazards regression model was fitted, and the covariates were considered significant if *P*<0.05. Multivariate adjusted analyses were used with sex, HCV genotype, age at infection, alcohol consumption, ALT and centre as covariates.

For the identification of interactions between *rs12979860* genotype and known risk factors for fibrosis progression, namely age, sex, alcohol consumption, ALT (a surrogate marker of necro-inflammation) and HCV genotype, we used multiple approaches to confirm the consistency of the results. These included stratified analyses to assess whether the *rs12979860* genotype OR across two levels of each of these clinical factors differed significantly: multiplicative interactions models and genotype-clinical factor joint effects^34^,^35^.

For continuous variables, the cohort was divided into two groups for the comparisons: age at time of biopsy (<40 and ≥40 years), ALT (<40 and ≥40 IU l^−1^), ALT (<71 and ≥71 IU l^−1^; the median of ALT in the cohort) and alcohol intake (none or <50 g daily and ≥50 g daily). For the categorical variables, the cohort was categorized as: sex (male or female) and (HCV genotype 3 or HCV non-genotype 3). Likelihood ratio tests were used to test for significance of OR differences between the clinical variable categories and a *P*-value below 0.05 (likelihood ratio test) was used to indicate a significant interaction.

Multiplicative interaction was evaluated by fitting a multiple logistic regression model with the main effects of risk factor and genotype, plus the interaction term. This was done for each of the known risk factors.

## Author contributions

M.E., J.G., D.B. and G.A. conceived the research. Enroling of patients, clinical phenotype data collation and sample acquisition/DNA preparation was performed by M.E., J.G., C.L., M.W.D., G.A., M.R.-G., T.B., G.J.D., H.L.Y.C, W.L.I., D.S., M.L.A., L.A.A., A.M., M.W., E.B., U.S., O.S., J.F., L.M., W.C., E.P., J.N., S.R., J.A., M.B., V.C.W., C.R., R.W., L.M., V.S., M.M., B.M., G.M., T.A., J.G., V.F. and J.R.J. Genotyping was performed by R.L. Histological analysis of tissues and scoring was conducted by D.M. Statistical analysis and interpretation of results was performed by M.E., J.G., A.H. and N.A. The manuscript was principally written and revised by M.E. and J.G. All authors critically reviewed the manuscript for important intellectual content and approved the final submitted manuscript.

## Additional information

**How to cite this article:** Eslam, M. *et al*. Interferon-λ rs12979860 genotype and liver fibrosis in viral and non-viral chronic liver disease. *Nat. Commun.* 6:6422 doi: 10.1038/ncomms7422 (2015).

## Supplementary Material

Supplementary Information Supplementary Tables 1-9

## Figures and Tables

**Table 1:** Univariate and multivariate analysis of factors associated with fibrosis stage ≥2 in 3,129 patients with CHC.

**Variables**	**Fibrosis <2**	**Fibrosis** ≥**2**	**Univariate analysis**	**Multivariate analysis**	
	**(*****n*****=1,584)**	**(*****n*****=1,545)**	***P-*****value**	**OR (95% CI)**	***P-*****value**
Age at time of biopsy (years)	42 (18–62)	47 (18–69)	<0.0001*	1.03 (1.02–1.05)	<0.0001
Male (%)	944 (47.7)	1036 (52.3)	<0.0001†	1.35 (1.19–1.59)	<0.0001

*Alcohol intake (%)*					
None or <50 g daily (%)	1373 (51.2)	1310 (48.8)	0.1†	—	—
≥50 g daily (%)	211 (47.3)	235 (52.7)			
					
*HCV genotype*					
HCV-3 (%)	231 (46.3)	268 (53.7)	0.03†	1.21 (1.01–1.48)	0.03
Non-HCV-3 (%)	1353 (51.4)	1277 (48.6)			
					
BMI (kg m^−2^)	25.3 (16–46)	26 (16–46)	<0.0001*	1.002 (0.999–1.004)	0.1
ALT (IU l^−1^)	60 (12–596)	86 (12–916)	<0.0001‡	1.003 (0.941–1.32)	0.2
AST (IU l^−1^)	45 (11–678)	64 (11–678)	<0.0001‡	1.19 (1.11–1.45)	0.001
ALP (IU l^−1^)	81 (10–902)	90 (14–1018)	0.001‡	1 (0.996–1.004)	0.9
Bilirubin (mg dl^−1^)	0.63 (0.1–4.71)	0.68 (0.1–4.72)	<0.0001‡	1.34 (0.85–2.1)	0.2
GGT (IU l^−1^)	41 (7–581)	61 (7–851)	<0.0001‡	1.14 (1.003–1.23)	0.01
Platelet (× 10^9^ l^−1^)	224 (54–577)	187 (43–674)	<0.0001‡	0.993 (0.990–0.996)	0.0001
HCV-RNA log_10_ (IU l^−1^)	5.92 (2.4–7.95)	5.9 (2.51–7.84)	0.8‡	—	—
					
*rs12979860*					
CC (%)	485 (43)	642 (57)	<0.0001†	1.63 (1.24–2.51)	<0.0001
CT/TT (%)	1099 (54.9)	903 (45.1)			
					
*Inflammation score*					
None/mild (%)	1138 (65.5)	637 (34.5)	<0.0001†	3.43 (3.13–4.22)	<0.0001
Moderate severe (%)	446 (31.5)	908 (68.5)			
					
*Steatosis degree*					
None/mild (%)	1295 (50.5)	1266 (49.5)	0.8†	—	—
Moderate severe (%)	289 (50.9)	279 (49.1)			

ALP, alkaline phosphatase; ALT, alanine aminotransferase; AST, aspartate aminotransferase; BMI, body mass index; CHC, chronic hepatitis C; CI, confidence interval; GGT, γ-glutamyl transpeptidase; OR, odds ratio.

Data are median and range or as %. Liver biopsy data are according to Metavir score. The OR was expressed as the risk of significant fibrosis per unit score change of the variables and per 10 IU l^−1^ increase in ALT, AST and ALP.

^*^Student’s *t*-test.

^†^Fisher’s exact test.

^‡^Mann–Whitney *U*-test.

**Table 2:** Stratification and interaction analyses for the association of *rs12979860* genotype.

	***rs12979860*****(Fibrosis** ≥**2/fibrosis <2)**				**Multiplicative interaction**
	**CC (*****n*****=1127)**	**CT/TT (*****n*****=2002)**	**OR (95% CI)**	***P-***value*	***P*****-value**
*Age at time of biopsy (years)*					
<40	230/228	207/508	2.47 (1.93–3.16)	0.01	0.02
≥40	433/236	675/612	1.66 (1.37–2.01)		
					
*Sex*					
Male	416/322	620/622	1.29 (1.07–1.55)	0.0002	0.01
Female	226/163	283/477	2.33 (1.82–2.99)		
					
*HCV genotype*					
HCV-3	166/92	102/139	2.45 (1.71–3.52)	0.009	0.1
HCV-non 3	476/393	801/960	1.45 (1.23–1.7)		
					
*ALT*					
<40 (IU l^−1^)	126/152	234/416	1.47 (1.1–1.96)	0.6	0.6
≥40 (IU l^−1^)	516/333	669/683	1.58 (1.32–188)		
					
ALT†					
<71 (IU l^−1^)	245/256	426/736	1.65 (1.33–2.04)	0.1	0.3
≥71 (IU l^−1^)	397/229	477/363	1.32 (1.06–1.63)		
					
*Alcohol intake*					
None or <50 g daily	620/335	690/1038	2.78 (2.36–3.28)	0.3	0.9
≥50 g daily	112/60	123/151	2.29 (1.54–3.39)		

ALT, alanine aminotransferase; CI, confidence interval; OR, odds ratio.

Stratification and interaction analyses for the association of *rs12979860* genotype, other clinical risk variables and liver fibrosis stage in the cohort of patients with chronic hepatitis C (*n*=3,129).

Data are as proportion by each category.

^*^P-value for differences between the OR for rs12979860 genotype within two strata of each clinical variable compared with each other. The OR are the result of univariate analysis. The interaction between the rs12979860 genotype and other clinical risk variables is quantified by the multiplicative measures of interaction.

^†^This stratification is based on median of ALT in the overall cohort.

**Table 3:** Univariate and Multivariate Analysis of Factors Associated with fibrosis stage >2 in 555 patients with chronic hepatitis B.

**Variables**	**Fibrosis ≤2**	**Fibrosis >2**	**Univariate analysis**	**Multivariate analysis**
	**(*****n*****=418)**	**(*****n*****=137)**	***P-*****value**	**OR (95% CI),** ***P-*****value**
Age at time of biopsy (years)	41.6±11.54	45.8±12.1	0.0001*	1.13 (1.09–2.31), 0.01
Male (%)	281 (67.2)	107 (78.1)	0.01†	—

*HbeAg*				
HbeAg positive (%)	259 (62)	81 (59.1)	0.6†	—
HbeAg negative (%)	159 (38)	56 (40.9)		
				
BMI (kg m^−2^)	22.99±3.4	24.4±4.4	0.0001*	—
ALT (IU l^−1^)	93.3±44.3	102±31.5	0.03‡	—
AST (IU l^−1^)	56±30.97	68.4±36.4	0.0001‡	1.03 (1.003–1.064), 0.03
Alkaline phosphatase (IU l^−1^)	78.7±25.5	86.3±32.2	0.007‡	—
GGT (IU l^−1^)	40.5±32.2	70.5±59.5	0.0001‡	1.014 (1.006–1.023) 0.001
Bilirubin (μmol l^−1^)	13.4±7.6	15.4±8.96	0.01‡	—
Albumin (g l^−1^)	44.4±4.06	42.96±4.8	0.002‡	—
Platelet (× 10^9^ l^−1^)	219.7±57.2	179.5±55.8	0.0001‡	0.989 (0.982–0.993), 0.001

*rs12979860*				
CC (%)	309 (73.9)	120 (87.6)	0.001†	2.75 (1.23–6.14), 0.001
CT/TT (%)	109 (26.1)	17 (12.4)		
HBV DNA (Log_10_, IU l^−1^)	5.96±2.2	6.6±2.04	0.003‡	1.68 (1.02–2.36), 0.001

ALP, alkaline phosphatase; ALT, alanine aminotransferase; AST, aspartate aminotransferase; BMI, body mass index; CHC, chronic hepatitis C; CI, confidence interval; GGT, γ-glutamyl transpeptidase; OR, odds ratio.

Data are as mean±s.d. or as %. Liver biopsy data are according to Metavir score. The OR was expressed as the risk of significant fibrosis per unit score change of the variables and per 10 IU l^−1^ increase in ALT, AST and ALP.

^*^Student’s *t*-test

^†^Fisher’s exact test.

^‡^Mann–Whitney *U*-test.

**Table 4:** Univariate and multivariate analysis of factors associated with fibrosis stage ≥2 in 488 patients with NAFLD.

**Variables**	**Fibrosis<2**	**Fibrosis**≥**2**	**Univariate analysis**	**Multivariate analysis**
	**(*****n*****=308)**	**(*****n*****=180)**	***P-*****value**	**OR (95% CI),** ***P-*****value**
Age at time of biopsy (years)	47.5±11.7	51.4±12.9	0.001*	1.15 (1.09–1.4) 0.01
Male (%)	153 (49.7)	99 (55)	0.2†	—
Diabetics (%)	68 (22.1)	74 (41.11)	<0.0001†	—
Hypertensive (%)	103 (33.44)	87 (48.33)	0.001†	1.56 (1.27–2.6) 0.001
BMI (kg m^−2^)	32.23±6.4	33.5±8.47	0.09*	—
ALT (IU l^−1^)	61.8±38.02	86.6±65.9	0.002‡	1.001 (0.992–1.01) 0.7
AST (IU l^−1^)	41.7±22.9	60.4±39.3	<0.0001‡	1.5 (1.06–1.84) 0.0001
GGT (IU l^−1^)	69.5±57.6	116.5±90.9	<0.0001‡	1.1 (0.98–1.3) 0.2
Platelet (× 10^9^ l^−1^)	251.4±62.6	221.4±84.9	<0.0001‡	0.995 (0.991–0.998) 0.004
Triglycerides (mmol l^−1^)	2.03±1.3	1.94±1.08	0.4‡	—
LDL-C (mmol l^−1^)	3.02±0.99	2.98±1.03	0.6‡	—
HDL-C (mmol l^−1^)	1.26±0.81	1.16±0.33	0.1‡	—
Blood glucose (mmol l^−1^)	5.9±2.5	6.7±2.5	0.001‡	—
Insulin (μU l^−1^)	14.9±10.8	20.9±15.7	<0.0001‡	—
HOMA-IR	3.8±3.4	6.1±4.9	<0.0001‡	1.3 (1.03–1.66) 0.001

*rs12979860*				
CC (%)	140 (45.5)	105 (58.3)	0.007†	1.66 (1.15–2.43) 0.006
CT/TT (%)	168 (54.5)	75 (41.7)		

HDL-C, high-density lipoprotein cholesterol; HOMA-IR, homeostasis model assessment-estimated insulin resistance; LDL-C, low-density lipoprotein cholesterol; NAFLD, non-alcoholic fatty liver disease.

Data are as mean±s.d. or as %. Owing to collinearity, only HOMA-IR was included in the model but not blood glucose and insulin and diabetes status. In another model excluding HOMA-IR, blood glucose and insulin levels, the adjusted OR for the presence of diabetes was (OR: 2.36, 95% CI: 1.65–3.77; *P*=0.0001). rs12979860 remained independently associated with Fibrosis ≥2 in this model (OR: 1.63, 95% CI: 1.14–2.45; *P*=0.006). The OR was expressed as the risk of significant fibrosis per unit score change of the variables and per 10 IU l^−1^ increase in ALT, AST and GGT.

^*^Student’s *t*-test.

^†^Fisher’s exact test.

^‡^Mann–Whitney *U*-test.

## References

[b1] Wynn A. Cellular and molecular mechanisms of fibrosis. J. Pathol. 214, 199–210 (2008) . 18161745 10.1002/path.2277 PMC2693329

[b2] Iredale J. P. Models of liver fibrosis: exploring the dynamic nature of inflammation and repair in a solid organ. J. Clin. Invest. 117, 539–548 (2007) . 17332881 10.1172/JCI30542 PMC1804370

[b3] Mehal W. Z., Iredale J. & Friedman S. L. Scraping fibrosis: expressway to the core of fibrosis. Nat. Med. 17, 552–553 (2011) . 21546973 10.1038/nm0511-552 PMC3219752

[b4] Romero-Gomez M., Eslam M., Ruiz A. & Maraver M. Genes and hepatitis C: susceptibility, fibrosis progression and response to treatment. Liver Int. 31, 443–460 (2011) . 21382156 10.1111/j.1478-3231.2011.02449.x

[b5] Patel K. . HLA class I allelic diversity and progression of fibrosis in patients with chronic hepatitis C. Hepatology 43, 241–249 (2006) . 16440356 10.1002/hep.21040

[b6] Knapp S. . Polymorphisms in interferon-induced genes and the outcome of hepatitis C virus infection: roles of MxA, OAS-1 and PKR. Genes Immun. 4, 411–419 (2003) . 12944978 10.1038/sj.gene.6363984

[b7] Ge D. . Genetic variation in IL28B predicts hepatitis C treatment-induced viral clearance. Nature 461, 399–401 (2009) . 19684573 10.1038/nature08309

[b8] Suppiah V. . IL28B is associated with response to chronic hepatitis C interferon-alpha and ribavirin therapy. Nat. Genet. 41, 1100–1104 (2009) . 19749758 10.1038/ng.447

[b9] Tanaka Y. . Genome-wide association of IL28B with response to pegylated interferon-alpha and ribavirin therapy for chronic hepatitis C. Nat. Genet. 41, 1105–1109 (2009) . 19749757 10.1038/ng.449

[b10] Eslam M. . *IFNL3* polymorphisms predict response to therapy in chronic hepatitis C genotype 2/3 infection. J. Hepatol. 61, 235–241 (2014) . 24768758 10.1016/j.jhep.2014.03.039

[b11] Prokunina-Olsson L. . A variant upstream of IFNL3 (IL28B) creating a new interferon gene IFNL4 is associated with impaired clearance of hepatitis C virus. Nat. Genet. 45, 164–171 (2013) . 23291588 10.1038/ng.2521 PMC3793390

[b12] Bochud P. Y. . IL28B alleles associated with poor hepatitis C virus (HCV) clearance protect against inflammation and fibrosis in patients infected with non-1 HCV genotypes. Hepatology 55, 384–394 (2012) . 22180014 10.1002/hep.24678

[b13] Noureddin M. . Association of IL28B genotype with fibrosis progression and clinical outcomes in patients with chronic hepatitis C: A longitudinal analysis. Hepatology 58, 1548–1557 (2013) . 23703931 10.1002/hep.26506 PMC3758382

[b14] Thompson A. J. . Genome wide-association study identifies IL28B polymorphism to be associated with baseline ALT and hepatic necro-inflammatory activity in chronic hepatitis C patients enrolled in the IDEAL study (Abstract). Hepatology 52, 1220A (2010) .

[b15] Marabita F. . Genetic variation in the interleukin-28B gene is not associated with fibrosis progression in patients with chronic hepatitis C and known date of infection. Hepatology 54, 1127–1134 (2011) . 21721028 10.1002/hep.24503

[b16] Di Marco V. . IL28B polymorphisms influence stage of fibrosis and spontaneous or interferon-induced viral clearance in thalassemia patients with hepatitis C virus infection. Haematologica 97, 679–686 (2012) . 22180419 10.3324/haematol.2011.050351 PMC3342968

[b17] Fabris C. . IL-28B rs12979860 C/T allele distribution in patients with liver cirrhosis: role in the course of chronic viral hepatitis and the development of HCC. J. Hepatol. 54, 716–722 (2011) . 21146242 10.1016/j.jhep.2010.07.019

[b18] Falleti E. . Role of interleukin 28B rs12979860 C/T polymorphism on the histological outcome of chronic hepatitis C: relationship with gender and viral genotype. J. Clin. Immunol. 31, 891–899 (2011) . 21647799 10.1007/s10875-011-9547-1

[b19] Abe H. . Common variation of IL28 affects gamma-GTP levels and inflammation of the liver in chronically infected hepatitis C virus patients. J. Hepatol. 53, 439–443 (2010) . 20576307 10.1016/j.jhep.2010.03.022

[b20] Garrett M. E. . IL28B rs12979860 is not associated with histologic features of NAFLD in a cohort of Caucasian North American patients. J. Hepatol. 58, 402–403 (2013) . 23063570 10.1016/j.jhep.2012.09.035 PMC3601946

[b21] Petta S. . IL28B and PNPLA3 polymorphisms affect histological liver damage in patients with non-alcoholic fatty liver disease. J. Hepatol. 56, 1356–1362 (2012) . 22314430 10.1016/j.jhep.2012.01.007

[b22] Poynard T. . Rates and risk factors of liver fibrosis progression in patients with chronic hepatitis C. J. Hepatol. 34, 730–739 (2001) . 11434620 10.1016/s0168-8278(00)00097-0

[b23] Ghany M. G. . Progression of fibrosis in chronic hepatitis C. Gastroenterology 124, 97–104 (2003) . 12512034 10.1053/gast.2003.50018

[b24] Ryder S. D., Irving W. L., Jones D. A., Neal K. R. & Underwood J. C. Progression of hepatic fibrosis in patients with hepatitis C: a prospective repeat liver biopsy study. Gut 53, 451–455 (2004) . 14960533 10.1136/gut.2003.021691 PMC1773967

[b25] Sato M. . Impact of IL28B genetic variation on HCV-induced liver fibrosis, inflammation, and steatosis: a meta-analysis. PLoS One 9, e91822 (2014) . 24637774 10.1371/journal.pone.0091822 PMC3956722

[b26] Villa E. . Reproductive status is associated with the severity of fibrosis in women with hepatitis C. PLoS One 7, e44624 (2012) . 22970270 10.1371/journal.pone.0044624 PMC3438179

[b27] Poynard T., Bedossa P. & Opolon P. Natural history of liver fibrosis progression in patients with chronic hepatitis C. The OBSVIRC, METAVIR, CLINIVIR, and DOSVIRC groups. Lancet. 349, 825–832 (1997) . 9121257 10.1016/s0140-6736(96)07642-8

[b28] Lawson A. . Hepatitis C virus-infected patients with a persistently normal alanine aminotransferase: do they exist and is this really a group with mild disease? J. Viral Hepatol. 17, 51–58 (2010) . 10.1111/j.1365-2893.2009.01148.x 19656289

[b29] Baran B. . Treatment failure may lead to accelerated fibrosis progression in patients with chronic hepatitis C. J. Viral Hepatol. 21, 111–120 (2014) . 10.1111/jvh.12127 24383924

[b30] Probst A. . Role of hepatitis C virus genotype 3 in liver fibrosis progression--a systematic review and meta-analysis. J. Viral Hepatol. 18, 745–759 (2011) . 10.1111/j.1365-2893.2011.01481.x 21992794

[b31] van der Meer A. J. . Association between sustained virological response and all-cause mortality among patients with chronic hepatitis C and advanced hepatic fibrosis. JAMA 308, 2584–2593 (2012) . 23268517 10.1001/jama.2012.144878

[b32] Lagging M. . Response prediction in chronic hepatitis C by assessment of IP- 10 and IL28B-related single nucleotide polymorphisms. PLoS One 6, e17232 (2011) . 21390311 10.1371/journal.pone.0017232 PMC3044738

[b33] Grebely J. . The effects of female sex, viral genotype, and IL28B genotype on spontaneous clearance of acute hepatitis C Virus infection. Hepatology 59, 109–120 (2014) . 23908124 10.1002/hep.26639 PMC3972017

[b34] Gomez-Santos L. . Inhibition of natural killer cells protects the liver against acute injury in the absence of glycine N-methyltransferase. Hepatology 56, 747–759 (2012) . 22392635 10.1002/hep.25694 PMC3378767

[b35] Suppiah V. . IL28B, HLA-C, and KIR variants additively predict response to therapy in chronic hepatitis C virus infection in a European Cohort: a cross-sectional study. PLoS Med. 8, e1001092 (2011) . 21931540 10.1371/journal.pmed.1001092 PMC3172251

[b36] Kumar V. . Genome-wide association study identifies a susceptibility locus for HCV induced hepatocellular carcinoma. Nat. Genet. 43, 455–458 (2011) . 21499248 10.1038/ng.809

[b37] Zhang H. . Genome-wide association study identifies 1p36.22 as a new susceptibility locus for hepatocellular carcinoma in chronic hepatitis B virus carriers. Nat. Genet. 42, 755–758 (2010) . 20676096 10.1038/ng.638

[b38] McClellan J. & King M. C. Genetic heterogeneity in human disease. Cell 141, 210–217 (2010) . 20403315 10.1016/j.cell.2010.03.032

[b39] Lee J. C. . Human SNP links differential outcomes in inflammatory and infectious disease to a FOXO3-regulated pathway. Cell 155, 57–69 (2013) . 24035192 10.1016/j.cell.2013.08.034 PMC3790457

[b40] Bibert S. . IL28B expression depends on a novel TT/-G polymorphism which improves HCV clearance prediction. J. Exp. Med. 210, 1109–1116 (2013) . 23712427 10.1084/jem.20130012 PMC3674704

[b41] McFarland A. P. . The favorable IFNL3 genotype escapes mRNA decay mediated by AU-rich elements and hepatitis C virus-induced microRNAs. Nat. Immunol. 15, 72–79 (2014) . 24241692 10.1038/ni.2758 PMC4183367

[b42] Smith K. R. . Identification of improved IL28B SNPs and haplotypes for prediction of drug response in treatment of hepatitis C using massively parallel sequencing in a cross-sectional European cohort. Genome Med. 3, 57 (2011) . 21884576 10.1186/gm273 PMC3238183

[b43] Sheahan T. . Interferon lambda alleles predict innate antiviral immune responses and hepatitis C virus permissiveness. Cell Host Microbe 15, 190–202 (2014) . 24528865 10.1016/j.chom.2014.01.007 PMC4104123

[b44] Raglow Z., Thoma-Perry C., Gilroy R. & Wan Y. J. IL28B genotype and the expression of ISGs in normal liver. Liver Int. 33, 991–998 (2013) . 23522062 10.1111/liv.12148 PMC7231429

[b45] American Diabetes Association. Diagnosis and classification of diabetes mellitus. Diabetes Care 27, s5–10 (2004) . 14693921 10.2337/diacare.27.2007.s5

[b46] Colloredo G., Guido M., Sonzogni A. & Leandro G. Impact of liver biopsy size on histological evaluation of chronic viral hepatitis: the smaller the sample, the milder the disease. J. Hepatol. 39, 239–244 (2003) . 12873821 10.1016/s0168-8278(03)00191-0

[b47] Bedossa P. & Poynard T. An algorithm for the grading of activity in chronic hepatitis C. The METAVIR Cooperative Study Group. Hepatology 24, 289 (1996) . 8690394 10.1002/hep.510240201

[b48] Kleiner D. E. . Design and validation of a histological scoring system for nonalcoholic fatty liver disease. Hepatology 41, 1313–1321 (2005) . 15915461 10.1002/hep.20701

[b49] Solé X., Guinó E., Valls J., Iniesta R. & Moreno V. SNPStats: a web tool for the analysis of association studies. Bioinformatics 22, 1928–1929 (2006) . 16720584 10.1093/bioinformatics/btl268

[b50] Lewis C. M. Genetic association studies: design, analysis and interpretation. Brief Bioinform. 3, 146–153 (2002) . 12139434 10.1093/bib/3.2.146

[b51] Akaike H. Fitting autoregressive models for prediction. Ann. Inst. Stat. Math. 21, 243–247 (1969) .

[b52] Yi Q., Wang P. P. & Krahn M. Improving the accuracy of long-term prognostic estimates in hepatitis C virus infection. J. Viral Hepatol. 11, 166–174 (2004) . 10.1046/j.1365-2893.2003.00484.x 14996352

