# Supplementary material for: Interferon-λ rs12979860 genotype and liver fibrosis in viral and non-viral chronic liver disease
Source: Nat Commun. 2015 Mar 5;6:6422. doi: 10.1038/ncomms7422 (PMC4366528; doi:10.1038/ncomms7422)
Supplement: Supplementary Information — Supplementary Tables 1-9 [file ncomms7422-s1.pdf]

Supplementary Table 1. The distribution of *IFNL rs12979860 and rs8099917* and Hardy-Weinberg equilibrium

**Genotype**	**Observed**	**Expected**	X^2	***P*-value***
**CHC**				
rs12979860 (n=3129)				
CC	1127	1145.8	1.98	0.1
CT	1533	1495.3		
TT	469	487.8		
rs8099917 (n=1997)				
TT	1138	1150.1	2.18	0.1
TG	755	730.8		
GG	104	116.1		
**CHB**				
rs12979860				
CC	429	425.6	1.77	0.1
CT	114	120.8		
TT	12	8.6		

NAFLD				
rs12979860				
CC	245	241.1	0.72	0.3
CT	196	203.8		
TT	47	43.1		

** P values were calculated by chi square test, P >0.05 indicates no deviation from Hardy-Weinberg equilibrium.*

Supplementary Table 2. Characteristics of the CHC cohort (n=3129)

Variables	Overall (n=3129)
**Age**	44.56±10.84<br>44 (18-69)
**Male (%)**	1980(63.3)
**BMI(Kg/m^2)**	26.23±4.62<br>25.64 (16-46)
**ALT (IU/L)**	98.6±88.3<br>71 (12-916)
**AST (IU/L)**	71.6±59.1<br>53 (11-678)
**GGT (IU/L)**	78.2±62.4<br>49 (7-851)
**Platelet (x10^9/L)**	212.4±70<br>208 (53-674)
**HCV-RNA log$_{10}$**	5.91±0.76<br>5.92 (2.4-7.95)
**HCV-genotype (%) 1, 2, 3, 4**	2290 (73.2), 232 (7.4), 499 (15.9), 108 (3.5)
**Liver fibrosis**	
None/mild	1584(50.6)
Moderate/severe	1545(49.4)
**Inflammation score**	
None/mild	1775(56.7)
Moderate/severe	1354(43.3)
***rs12979860* (%)**	
CC (%)	1127 (36)
CT/TT (%)	2002 (64)
**Alcohol history (%)**	
None or less than 50 g/daily (%)	2683 (85.7)

≥ 50 g/daily (%)	446(14.3)
**Steatosis degree**	
None/mild (%)	2561(81.8)
Moderate severe (%)	568(18.2)

Data are mean and standard deviation (SD), median and range or as %. Liver biopsy data are according to Metavir score.

Supplementary Table 3. Odds ratio, Akaike's Information Criterion (AIC) and Bayesian Information Criterion (BIC) values for various rs12979860 genetic models of inheritance.

Genetic Model	OR	P-value*	Model Fit
			AIC
**Codominant model**			
TT	ref	-	4301.5
CT	1.11 (0.9-1.73)	0.3	
CC	1.74 (1.4-2.17)	<0.0001	
**Dominant model**			
CT+TT	ref	-	4300.6
CC	1.63 (1.24-2.51)	<0.0001	
**Recessive model**			
TT	ref	-	4332.4
CC+CT	1.34 (1.1- 1.64)	<0.003	
**Overdominant**			
CT	ref	-	4325.2
TT+CC	1.33 (1.15-1.53)	<0.0001	
**Log Additive model**			
-	1.36(1.23-1.51)	<0.0001	4304.7

*Odds ratio (OR) was calculated by logistic regression BIC, Bayesian information criterion; AIC, Akaike information criterion. Lower AIC and BIC values indicate a better fit. * Logistic regression analysis.*

Supplementary Table 4: Univariate and multivariate analysis of *rs12979860* Genotype associations With Clinical and Laboratory Characteristics in 3129 patients with Chronic Hepatitis C Patients

Variables	CC	CT/TT	Univariate Analysis	Multivariate Analysis	
	(n=1127)	(n=2002)	*P* value	OR (95% CI)	*P* value
**Age at time of biopsy (yrs)**	43.3 (18-69)	44.1 (18-69)	0.5†	-	-
**Male Gender (%)**	738 (37.3)	1242 (62.7)	0.06*	-	-
**Alcohol intake (%)***			0.2*	-	-
None or less than 50 g/daily (%)	955(35.6)	1728(64.4)			
≥ 50 g/daily (%)	172(38.6)	274(61.4)			
**HCV genotype**			<0.0001*	1.78(1.28-2.48)#	<0.0001
1, (%)	736 (32.1)	1554 (67.9)			
2, (%)	96 (41.4)	136 (58.6)			
3, (%)	258 (51.7)	241 (48.3)			
4, (%)	38 (35.2)	70 (64.8)			
**Body Mass Index (Kg/m^2)**	25.6 (16-46)	25.7 (16-46)	0.9†	-	-
**ALT (IU/L)**	88 (12-916)	65 (12-693)	<0.0001‡	1.2 (1.1-1.44)	<0.0001
**AST (IU/L)**	63 (11-678)	49.7 (11-678)	<0.0001‡	1.002 (0.992-1.003)	0.3
**Alkaline phosphatase (IU/L)**	82 (12-1018)	86 (10-902)	0.1‡	-	-
**Bilirubin (mg/dl)**	0.7 (0.1-4.72)	0.64 (0.1-4.27)	0.001‡	1.2 (0.603-1.37)	0.2
**GGT (IU/L)**	54 (7-851)	41 (7-851)	<0.0001‡	1.004 (1.002-1.007)	0.001
**Platelet (x10^9/L)**	203 (54-577)	210 (53-674)	0.002‡	0.996 (0.993-0.999)	0.007
**HCV-RNA $\log_{10}$ (IU/ml)**	5.92 (2.51-7.84)	5.9 (2.4-7.95)	0.7‡	-	-

Data are as median and range or as %. Liver biopsy data are according to Metavir score. # HCV-3 vs. non HCV-3. The odds ratio was expressed as the risk of significant fibrosis per unit score change of the variables and per 10 IU/L increase in ALT, AST and ALP. †Student t test; ‡Mann-Whitney U test. * χ^2 and Fisher-exact tests.

Supplementary Table 5. Univariate and multivariate analysis of factors associated with fast fibrosis progression rates in 1312 patients with Chronic Hepatitis C

Variables	Fibrosis progression rate (fibrosis units/years)			Multivariate Analysis	
	<0.076 (n=647)	≥0.076 (n=665)	*P* value	OR (95% CI)	*P* value
**Age at time of biopsy (yrs)**	43 (18-69)	46.1 (18-69)	< 0.0001†	1.19 (1.15-1.33)	<0.0001
**Age at infection (yrs)**	19 (1-60)	23 (1-60)	<0.0001†	1.14 (1.11-1.27)	<0.0001
**Male Gender (%)**	392(46.1)	460 (53.9)	0.001	1.25 (1.16-1.83)	0.001
**Alcohol intake (%)**					
None or less than 50 g/daily (%)	477(51)	459(69)	0.06*	-	-
≥ 50 g/daily (%)	170(45.2)	206(54.8)			
**HCV genotype**					
HCV-3 (%)	98(42.4)	133(57.6)	0.02*	1.34 (0.86-2.08)	0.1
Non-HCV-3 (%)	549(50.8)	532(49.2)			
**Body Mass Index (Kg/m^2)**	26 (16-46)	25.6 (16-46)	0.4‡	-	-
**ALT (IU/L)**	66 (12-842)	93 (18-900)	<0.0001‡	0.997 (0.993-1.003)	0.2
**AST (IU/L)**	48 (12-405)	66 (14-471)	<0.0001‡	1.1 (1.02-1.2)	0.001
**Alkaline phosphatase (IU/L)**	78 (15-416)	81 (18-425)	0.1‡	-	-
**GGT (IU/L)**	44 (7-747)	62 (9-616)	<0.0001‡	1.04 (1.01-1.09)	0.001
**Platelet (x10^9/L)**	221 (81-674)	206 (56-497)	<0.0001‡	0.995 (0.992-0.998)	<0.0001
rs12979860					
CC (%)	204 (42.1)	281(57.9)	0.0001*	1.58 (1.26-1.99)	<0.0001
CT/TT (%)	443(53.6)	384(46.4)			
**HCV-RNA log$_{10}$**	5.84 (2.92-7.84)	5.9 (2.98-7.84)	0.1‡	-	
**Inflammation score**					
None/mild (%)	446(60.2)	295(39.8)	0.0001*	2.78 (2.22- 3.48)	< 0.0001
Moderate severe (%)	201(35.2)	370(64.8)			
**Steatosis degree**					

None/mild (%)<br>Moderate severe (%)	563(49.6)<br>84(47.5)	572(50.4)<br>93(52.5)	0.6*	-	-

*Data are as median and range or as %. Liver biopsy data are according to Metavir score. The odds ratio was expressed as the risk of significant fibrosis per unit score change of the variables and per 10 IU/L increase in ALT, AST and ALP. †Student t test; ‡Mann-Whitney U test.*Fisher-exact test.

Supplementary Table 6. Baseline characteristics of the sub-cohort of chronic hepatitis C with paired liver biopsies

Variables	HCV paired biopsies sub-cohort (n=106)
**Age (yrs)**	42±9
**Male (%)**	62 (58.4)
**BMI(Kg/m^2)**	26.4±4.5
**ALT (IU/L)**	112.7±92.5
**AST (IU/L)**	74.5±58.2
**GGT (IU/L)**	75.2±62.3
**Platelet (x10^9/L)**	221.1±68.1
**HCV-RNA log$_{10}$**	5.7±0.71
**HCV-genotype(%) 1, 2, 3**	61(57.6), 10(9.4), 35(33)
**Time between biopsies**	6.01 (2.36-9.45)
**Liver fibrosis**	
Metavir fibrosis stage (F0-F2)	88 (83)
Metavir fibrosis stage (F3)	18 (17)
**Inflammation score**	
Metavir grade (A0-A1)	85(80)
Metavir grade (A2)	21(20)
***rs12979860* (%)**	
CC (%)	44 (41.5)
CT/TT (%)	62 (58.5)
***rs8099917* (%)**	
TT (%)	60 (56.6)
TG/GG (%)	46 (43.4)

Data are mean and SD, median and range or as %. Liver biopsy data are according to Metavir score.

**Supplementary Table 7.** ***IFNL rs12979860 and rs8099917*** **genotypes association with changes in hepatic inflammation ≥2 points, any fibrosis progression and fibrosis progression ≥2 points in 106 patients with paired liver biopsies**					
**Variables**	**Changes in hepatic inflammation < 2 (n=38)**	**Changes in hepatic inflammation ≥ 2 (n=68)**	**P value***	**Multivariate Analysis**	
				**OR (95% CI)**	**P value#**
rs12979860					
CC (%)	8 (21)	36(53)	0.002	2.21(1.69-4.32)	0.002
CT/TT (%)	30 (79)	32(47)			
rs8099917					
TT (%)	11(29)	49(72)	<0.0001	4.33(2.62-7.24)	<0.0001
TG/GG (%)	27(71)	19(28)			
**Variables**	**No fibrosis progression (n=58)**	**Any fibrosis progression (n=48)**	***P*** **value**	**Multivariate Analysis**	
				**OR (95% CI)**	***P*** **value**
rs12979860					
CC (%)	20(34)	24(50)	0.1	-	-
CT/TT (%)	38(66)	24(50)			
rs8099917					
TT (%)	27(47)	33(69)	0.03	2.52(1.13-5.61)	0.02
TG/GG (%)	31(53)	15(31)			
**Variables**	**Changes in hepatic fibrosis < 2 (n=73)**	**Changes in hepatic fibrosis ≥ 2 (n=33)**	***P*** **value**	**Multivariate Analysis**	
				**OR (95% CI)**	***P*** **value**
rs12979860					
CC (%)	26(36)	18(55)	0.08	-	-
CT/TT (%)	47(64)	15(45)			
rs8099917					
TT (%)	36(49)	24(73)	0.03	2.74(1.12-6.69)	0.02
TG/GG (%)	37(51)	9(27)			

*Fisher-exact test. # Multiple logistic regression analysis

Supplementary Table 8. Characteristics of the chronic hepatitis B cohort

Variables	HBV Cohort (n=555)
**Age (yrs)**	42.6±11.9
**Male (%)**	388(69.3)
**BMI(Kg/m^2)**	23.09±6.2
**ALT (IU/L)**	93.98±81.5
**AST (IU/L)**	59.7±48.06
**GGT (IU/L)**	49.05±40.5
**Platelet (x10^9/L)**	210.5±58.5
**HBV DNA (Log$_{10}$ IU/mL)**	5.6±1.35
**HbeAg positive (%)**	215(38.7)
**Liver fibrosis**	
None/mild	284(51.1)
Moderate/severe	271(48.9)
**Inflammation score**	
None/mild	134(23.9)
Moderate/severe	421(75.2)
***rs12979860* (%)**	
CC (%)	429(77.3)
CT/TT (%)	126(22.7)

Data are as mean ± standard deviation or as %. Liver biopsy data are according to Metavir score.

Supplementary Table 9. Characteristics of the NAFLD cohort

Variables	NAFLD Cohort (n=488)
**Age(yrs)**	50±12.4
**Male (%)**	252(51.6)
**BMI(Kg/m^2)**	23.97±7.6
**Hypertensive (%)**	190 (38.9)
**Diabetics (%)**	142(29.1)
**ALT (IU/L)**	71.8±51.2
**AST (IU/L)**	50.5±33.8
**GGT (IU/L)**	89±66
**Platelet (x10^9/L)**	237.2±74.9
**HOMA-IR**	4.6±2.1
**Triglycerides, mmol/L**	1.99±1.5
**HDL-cholesterol, mmol/L**	1.2±0.66
**LDL-cholesterol, mmol/L**	2.99±1.04
**Liver fibrosis**	
None/mild	308(63.1)
Moderate/severe	180(36.9)
**Inflammation score**	
None/mild	418(88)
Moderate/severe	57(12)
***rs12979860* (%)**	
CC (%)	245(50.2)
CT/TT (%)	243(49.8)

Data are as mean ± standard deviation or as %.
